# Applying disease risk analysis for conservation translocations in Argentina: A case study on marsh deer (*Blastocerus dichotomus*)

**DOI:** 10.1371/journal.pone.0322878

**Published:** 2025-05-20

**Authors:** Guillermo M. Wiemeyer, Marcela M. Uhart, Lucía Rodríguez Planes, Virginia Rago, Eliana Carolina Guillemi, Elizabeth Chang Reissig, Mariana Raño, Dante Di Nucci, Pablo M. Beldomenico, Silvana Peker, Laura Geffner, M. Marcela Orozco

**Affiliations:** 1 Instituto de Ecología, Genética y Evolución de Buenos Aires (IEGEBA-CONICET), Ciudad Autónoma de Buenos Aires, Argentina; 2 Fundación Caburé-í, Ciudad Autónoma de Buenos Aires, Argentina; 3 Grupo de Investigaciones en Biología de la Conservación (INIBIOMA, CONICET-Universidad Nacional del Comahue), San Carlos de Bariloche, Argentina; 4 Karen C. Drayer Wildlife Health Center, School of Veterinary Medicine, University of California, Davis, California, United States of America; 5 Dirección Regional Patagonia Austral-Administración de Parques Nacionales, Instituto de Ciencias Polares Ambiente y Recursos Naturales, Universidad Nacional de Tierra del Fuego, Ushuaia, Argentina; 6 Instituto de Investigaciones en Biodiversidad y Medioambiente (INIBIOMA, CONICET-UNCo), Subsede Junín de los Andes, Neuquén, Argentina; 7 Instituto de Agrobiotecnología y Biología Molecular (IABIMO) INTA-CONICET, Buenos Aires, Argentina; 8 Instituto de Investigaciones Forestales y Agropecuarias Bariloche (IFAB; CONICET-INTA), San Carlos de Bariloche, Río Negro, Argentina; 9 Dirección Regional Noreste Argentino-Administración de Parques Nacionales, Corrientes Capital, Argentina; 10 Fundación de Historia Natural Félix de Azara, Centro de Rescate, Rehabilitación y Recría de Fauna Silvestre Güirá Oga, Puerto Iguazú, Misiones, Argentina; 11 Laboratorio de Ecología de Enfermedades, Instituto de Ciencias Veterinarias del Litoral (UNL-CONICET), Esperanza, Santa Fe, Argentina; 12 Dirección de Biodiversidad, Subsecretaría de Ambiente, Ciudad Autónoma de Buenos Aires, Argentina; 13 Coordinación de Zoonosis, Dirección Nacional de Control de Enfermedades Transmisibles, Ministerio de Salud de la Nación, Ciudad Autónoma de Buenos Aires, Argentina; Instituto Oswaldo Cruz, BRAZIL

## Abstract

Disease risk management is essential for conservation translocations to prevent inadvertent pathogen introduction affecting human, animal and ecosystem health. Wildlife Disease Risk Analysis (DRA) is a recognized framework for addressing health hazards in translocations. However, DRA is not mandatory nor voluntarily applied in Argentina, despite increasing wildlife translocations. To test and adapt DRA to the local context, we performed a simplified DRA for the hypothetical translocation of marsh deer (*Blastocerus dichotomus*) between two protected areas, Iberá and El Impenetrable National Parks. A multidisciplinary team applied the main phases of DRA, problem description, hazard identification, risk assessment and mitigation, to this scenario. Out of 61 potential hazards identified, including pathogens and management issues, 14 priority hazards were highlighted using a paired risk prioritization tool. Of these, 66% have zoonotic potential. Presence of *Ehrlichia chaffeensis* at the source (Iberá) but not the destination park signalled unacceptable risk under a One Health perspective. All other hazards, including pathogens, stress and seasonal factors, were considered manageable through strategic planning and mitigation actions. This study represents the first application of DRA to conservation translocations in Argentina, in a context of data and resource limitations. Strengthening baseline information and stakeholder engagement would enhance its utility. DRA findings should inform broader ecological evaluations to assess feasibility and relevance of translocations. We advocate for the integration of DRA into conservation planning in Argentina and South America, even under suboptimal conditions.

## 1 Introduction

Healthy ecosystems play a critical role in biodiversity conservation, climate change mitigation, and providing essential services for humanity [1]. However, human activity is leading to the degradation, modification, and fragmentation of ecosystems, upsetting natural processes that maintain ecosystem functionality [2–4]. Different mitigation strategies have been proposed to restore the lost ecological function, and each of them has advantages and disadvantages. However, given the complexity of the task, there are no perfect interventions and risk of collateral-effects is never zero [5–7]. One of several conservation tools applied when species diversity has been affected is restoring populations and ecological interactions to recover ecosystem functionality via wildlife translocations [8–10], the success or failure of which is influenced by a multitude of factors that have been extensively discussed and evaluated [11,12].

Wildlife usually hosts a diverse and complex micro and macroparasite community [13], which gets translocated together with the main host. The potential disruption of this host-parasite relationship can produce variable outcomes for hosts, parasites, environment, and can also represent a risk to human health [14,15]. Notwithstanding, pathogen spread has received limited attention in translocation efforts [16,17]. Moreover, even when health management can prevent disease transmission [18], the latter is often overlooked during the planning and implementation phases of translocations. A retrospective review of vertebrate translocation projects over a 13-year period found that 24% of these projects omitted disease screening, and only 22% included post-release follow-up to monitor disease-related mortality [19]. In a more recent review of conservation translocations, 61% of case studies (n = 180/295) mentioned at least one preemptive pre- and/or post-release health management measure being implemented [20], but only 4% reported the inclusion of disease-related risk assessments. Thus, while there has been a positive shift in recent years, significant gaps persist and holistic methodologies are infrequently implemented [21]. Despite increased attention, improving wildlife translocation through the implementation of disease risk management strategies is fundamental given the risks they pose to human, animal and ecosystem health.

**Fig 1:**
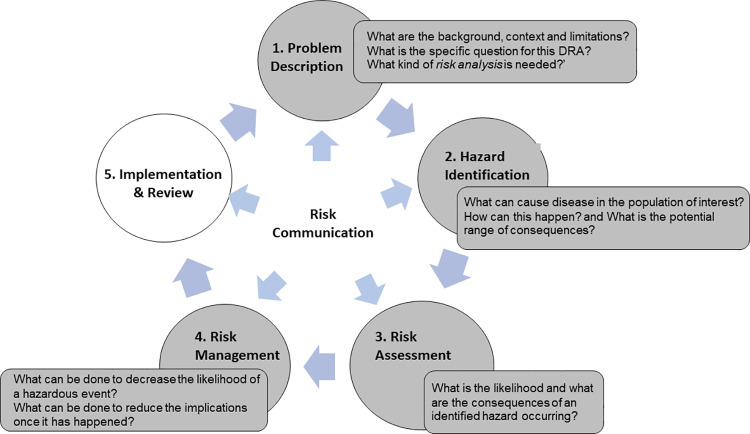
Five basic concatenated steps are needed for an iterative DRA process, articulated by fluid risk communication. Steps 1 to 4 (painted in grey) were addressed in this study. Each step presents guiding questions to illustrate the main content addressed (Adapted from Jakob-Hoff [22]).

Wildlife Disease Risk Analysis (DRA) [22] is a structured, evidence-based approach to managing disease risks for wildlife, which can be applied in the context of conservation translocations. This framework has been developed by the World Organisation for Animal Health (WOAH, former Office International des Epizooties-OIE), and the International Union for Conservation of Nature (IUCN). The analysis involves five basic steps interconnected through risk communication: 1) problem description, 2) hazard identification, 3) risk assessment, 4) risk management, and 5) implementation and review [22] (Fig 1). DRA acknowledges uncertainty, identifies information gaps and integrates diverse fields of expertise, recognizing their importance for the effective communication and collaboration across disciplines, cultures, and project partners. This approach ensures that risk assessment is based on the best-available knowledge, incorporates the interests of all partners and stakeholders, and yields risk management strategies that are widely accepted and feasible [23,24]. DRA outcomes help in effective implementation of participatory, science-based conservation strategies [25] to establish public policies for the conservation of the species through National Conservation Action Plans.

While DRA is recognized as an effective tool for addressing wildlife health issues for translocations in a structured mode, its adoption and implementation varies significantly across different regions and countries. DRA is commonly used in wildlife translocations in North America [24,26], Europe [16,23] and Oceania [27–29]. In contrast in Asia, Africa, and South America, DRA is rarely applied and reported.

South America is one of the world’s most significant biodiversity hotspots [30]. While at the landscape level it remains less impacted than other parts of the world [31], translocations are becoming increasingly common to mitigate the effects of habitat loss and fragmentation on species abundance and distribution [8]. A particular type of conservation translocation is oriented to restoring ecosystem functionality through reintroduction of locally extinct species [8]. Despite the expansion of such practices in Argentina [32], ethical and technical aspects of this type of ecological restoration have recently triggered considerable scientific debate in this country [33–36].

From a health risk perspective, a relevant weakness in current conservation translocation methodology in Argentina is the fact that beyond quarantine and veterinary care for translocated individuals, a broader health risk assessment, such as DRA, is neither requested by authorities nor voluntarily applied as best practice [35]. Illustrative of this shortcoming are some failed reintroduction attempts linked to diseases not anticipated because of the lack of risk analysis. For example, the reintroduction of tapir (*Tapirus terrestris*) to Iberá (Corrientes, Argentina) was initiated in 2016 from captive stock but was forced to stop two years later due to severe disease and death caused by the protozoan *Trypanosoma evansi* [37]. This parasite is endemic in Iberá with the abundant capybara (*Hydrochoerus hydrochaeris*) as the main reservoir [38], posing a known risk. The failure to address this risk led to preventable losses, ultimately requiring the recapture of the affected tapirs and the termination of the project.

Given the increasing number of translocation initiatives in Argentina, national authorities have identified the need to integrate more robust risk assessment methodologies into these projects. To this end, a joint fellowship from the National Parks Administration of Argentina and CONICET enabled the application of the DRA framework to a real case study presented here.

To avoid repetition of flawed attempts with potential trickling consequences for entire ecosystems, and to lay the groundwork for future comprehensive assessments, we performed a simplified risk assessment exercise under a hypothetical scenario, adapting the existing DRA tool to the local settings of Argentina. We used the translocation of marsh deer (*Blastocerus dichotomus)* between two protected areas in Argentina, Iberá National Park (Iberá) to El Impenetrable National Park (Impenetrable) as a case study. This scenario was based on a real project that did not include DRA and was eventually rejected by regulatory agencies in 2023, illustrating the necessity of incorporating this methodology into conservation translocations.

With the aim of empowering local regulatory agencies with appropriate tools and of enhancing regional expertise in wildlife translocation DRA, we present the outcome of a simplified DRA based on the WOAH/IUCN framework (Fig 1).

## 2 Materials and methods

### 2.1. Species and study site

This was a tabletop study, and did not include fieldwork or animal handling. Thus, under Argentina legislation, it did not require specific permits.

**Fig 2:**
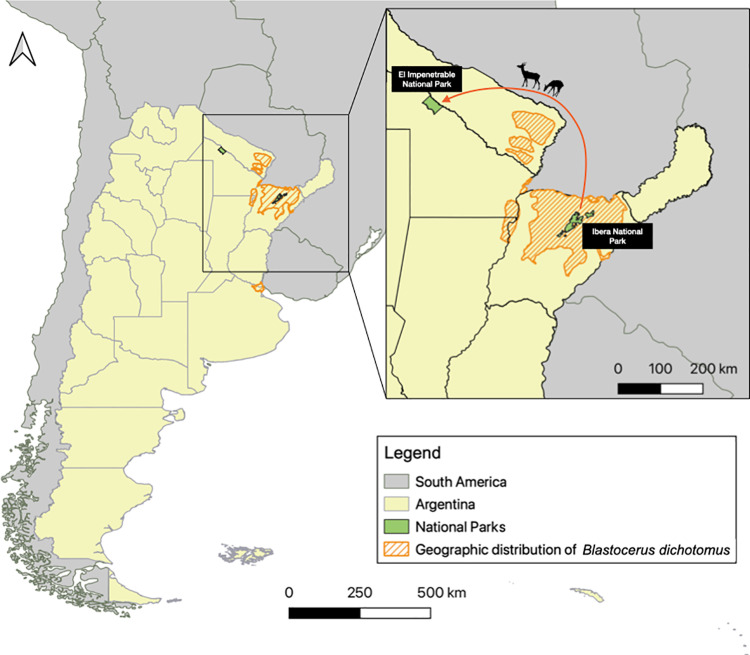
Hypothetical scenario for a marsh deer translocation case study. The map shows the source, Iberá National Park, and the destination, El Impenetrable National Park, illustrating the areas involved in the translocation. The arrow represents a conceptual depiction of the movement of the deer between these sites and is for illustrative purposes only. It does not correspond to the actual terrestrial route or precise path taken by the animals but is meant to highlight the general direction of movement. Map recreated in QGIS using geospatial layers from Instituto Geográfico Nacional de la República Argentina (IGN), Categorización de los Mamíferos de la Argentina, MAyDS & SAREM (CMA), and Sistema de Información sobre Biodiversidad – Administración de Parques Nacionales (SIB). Source: Instituto Geográfico Nacional (data accessed from https://www.ign.gob.ar/NuestrasActividades/InformacionGeoespacial/CapasSIG), Pereira et al. (2019), MAyDS & SAREM (data accessed from http://cma.sarem.org.ar), and SIB (https://sib.gob.ar/institucional/cartografia-parques-nacionales). Silhouettes of deer taken from http://www.philopic.org, freely available for reuse under Creative Commons licenses.

   

Our target species is the marsh deer. Marsh deer are the largest South American deer species and occur in marshy habitats south of the Amazon River into Argentina [39], Brazil, Paraguay, Peru, and Bolivia [40]. Generally solitary, aggregations of up to six animals have been reported during floods [41]. It is globally listed as vulnerable by the IUCN [42]. In Argentina, the species was categorized as vulnerable in the 2019 Red List of Argentine Mammals [43], with a projected 30% reduction in population size (over 15 years or 3 generations) due to habitat modification, poaching impact and extraordinary floods exacerbated by climate change [43]. Subsequently, it was listed as threatened by the Ministry of Environment and Sustainable Development in 2021 [44]. Habitat loss (due to wetland draining, forestry or urbanisation) and poaching are the main current threats. Deer aggregation in elevated land patches during flooding is associated with increased mortality due to illegal hunting, malnutrition, infectious diseases and extreme temperatures [45,46]. Its in-country distribution is fragmented into at least four subpopulations (Fig 2). While it is postulated that the Iberá subpopulation recovered significantly up to 2010 [47], multiple winter mass mortality events have affected these populations in the last decade [45,46]. Such episodes were documented, revealing associations between the winter season and poor body condition, high tick loads, infection with vector-borne pathogens, and presence of harmful gastrointestinal parasites [45,46].

The destination area in our scenario is El Impenetrable National Park (Impenetrable), created in 2014 to protect 128,000 hectares of the declining dry forests of the Dry Chaco ecoregion [48]. The Park is located in northwestern province of Chaco and comprises dry forest plains with a marked seasonal climate and a median temperature of 24 °C. The climate is semi-tropical continental, characterized by a marked continentality and high temperatures throughout the year, with peaks above 40 °C in any month and summer exceptionally reaching above 45 °C. In winter, minimum temperatures can drop below 5 °C. Average annual precipitation is around 400–800 mm falling mostly in October-April, with slight variations between the eastern and western areas of the park [49]. The Park is bounded to the north and south by two rivers, the Bermejo and the Bermejito. No people inhabit the Park, and the livestock burden is low, limited to stray cattle from neighbouring ranch outposts, but there are also feral donkeys and horses [49]. Poaching occurs, but its extent is unknown. Even though some naturalists mention isolated records of marsh deer presence in neighbouring areas [50], there is neither consensus nor historic record of its presence within Impenetrable territory.

The source area in our scenario is Iberá National Park (Iberá), created in 2018 to protect 195,000 hectares of the Iberá Wetlands ecoregion [48]. The Park is located in central-northern province of Corrientes, and is composed of a mosaic of different habitats, such as marshes, lagoons, streams, rivers, savannas, grasslands, and thorny and humid forests [51,52]. The climate of Iberá is subtropical without a dry season, a median temperature of 20 °C, and medial annual precipitation of 1500 mm [53]. The main productive activities in the region are agriculture, tourism and extensive cattle ranching [54]. It is currently home to the largest marsh deer subpopulation in Argentina [43] estimated at 8900 individuals (95% CI = 6740–11658) between 2006 and 2008 by De Angelo and colleagues [47].

### 2.2. The simplified DRA exercise used in this study

We applied a simplified DRA based on the WOAH/IUCN framework, including only the first four steps: 1) problem description, 2) hazard identification, 3) risk assessment (initially semi-quantitative and then qualitative), and 4) risk management. The process relied on discussion rounds among a multi-disciplinary team of coauthors, including epidemiologists, eco-epidemiologists, wildlife biologists, wildlife veterinarians, One Health practitioners, physicians, animal health government officers, environmental agency officers and national parks biologists. We conducted activities virtually, enabling participation of a geographically dispersed team, while maintaining a structured workflow, with a few specific scheduled online meetings.

#### 2.2.1 Step 1. Problem description.

During a first scheduled online meeting (3hs) in August 2023, and following the WOAH/IUCN DRA framework [22], we summarized background knowledge and context and agreed on the problem description with the first author acting as facilitator. Regarding the definition of populations of interest, it is important to clarify that the term “populations” was not used in a strict ecological context but in accordance with the mentioned framework to reference both a group of translocated deer as well as the community of different wildlife species receiving those deer. After identifying the overall goal, objectives and acceptable risk, we defined the scope and focus of the DRA, explicitly stating assumptions and limitations to allow planning of the following steps (Table 1). For this exercise, we selectively focused on disease hazards, including infectious and parasitic diseases, as well as diseases with metabolic or nutritional etiologies. Ecological background (e.g., historical occurrence in the area) or other hazards unrelated to disease (e.g., trauma, anesthesia-related complications, stress, genetic fitness, etc.), as well as overall feasibility, were beyond the scope of this study.

**Table 1:** Disease risk analysis problem description.

DRA Questions: What is the risk of disease transmission among translocated marsh deer, wildlife, livestock, and people in and around Impenetrable? How can it be mitigated?	
Goal	Objectives
To translocate healthy marsh deer to establish a self-sustaining population without introducing new pathogens or impacting wild populations.	1. Identify and minimize health risks for translocated deer, wildlife, and domestic animals in and around Impenetrable.
	2. Prevent the introduction of new pathogens or vectors into Impenetrable.
	3. Reduce exposure of staff, neighbors and park visitors to zoonotic diseases.
**Populations of interest**	
1-Marsh deer potentially translocated from Iberá to Impenetrable.	
2-Wildlife in and around Impenetrable that could transmit pathogens and vectors to translocated deer, or become infected with pathogens carried by translocated deer.	
3-Humans will be considered only in terms of zoonotic diseases despite not being a primary population of interest.	
**Acceptable risk:** Ensure translocation does not increase pathogen prevalence, vector loads, or mortality rates beyond expected levels for translocated deer, Impenetrable wildlife, neighboring animals, or staff.	
**Focus**: Infectious and non-infectious diseases that circulate among the populations of interest, potentially affecting them.	
**Assumptions**	
1- Wildlife in Impenetrable may harbor pathogens for translocated deer and act as hosts for transmissible agents.	
2-Livestock in or around Impenetrable may carry pathogens affecting translocated deer.	
3-Translocated deer may introduce novel pathogens to Impenetrable affecting local animals and humans.	
4-Translocated deer may interact with wildlife and domestic animals beyond protected area boundaries.	
**Limitations**	
1-Information gaps exist regarding disease prevalence and epidemiology among populations of interest.	
2-Non-medical factors such as feral dogs or illegal hunting are excluded from this analysis to focus on diseases.	
3-Entry of neighboring cattle into Impenetrable is poorly controlled and difficult to assess.	
4-Capture, anesthesia, and translocation risks can be minimized but not eliminated, especially challenging for large deer in drug-restricted regions like Argentina	

Problem description used to frame a wildlife Disease Risk Analysis (DRA) for the translocation of marsh deer (*Blastocerus dichotomus*) from Iberá to Impenetrable in Argentina. This framework includes the driving question, goal, objectives, populations of interest, focus, acceptable risk, assumptions and limitations for this exercise.

#### 2.2.2 Step 2. Hazard identification.

**Fig 3:**
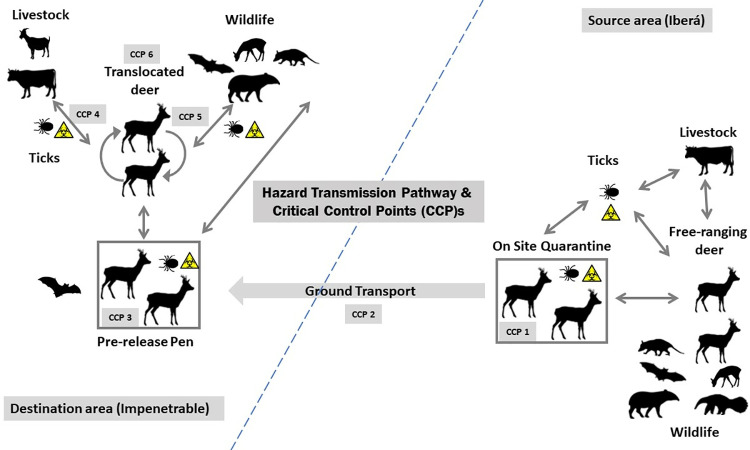
Hazard Transmission Pathway(s) and Critical Control Points (CCPs), identified for a hypothetical scenario of Marsh deer ground translocation from Source area (Iberá) to Destination area (Impenetrable) in Argentina and eventual contact with other animals. CCP1: On-Site Quarantine; CCP2: Ground Transport; CCP3: Pre-release Pen; CCP4: Transmission from/to translocated deer-livestock; CCP5: Transmission from/to translocated deer-wildlife; CCP6: translocated deer monitoring. Boxes represent confinement in human-made structures. Ticks with biohazard warning represent their plausible role as disease vectors. Animal silhouettes were taken from http://www.philopic.org, freely available for reuse under Creative Commons licenses and biohazard sign from http://www.openclipart.org with public domain.

 

To assist with hazard identification, we developed a schematic map of the hazard transmission pathway to identify geographical and temporal patterns of hazards exposure and potential consequences (Fig 3). We constructed a list of potential hazards using a shared online document, during a four-week period of asynchronic individual work. For this purpose, we revised and compiled a total of 175 documents, including published and unpublished data, book chapters, peer-reviewed scientific articles, agency reports, conferences proceedings, and theses (references available in S1). Diseases considered were primarily those affecting marsh deer in Argentina, but we also included diseases reported via surveillance, morbidity/mortality reports at source and destination Parks and surrounding areas, focusing more broadly on livestock and other domestic animals, wildlife, and humans (the latter only for zoonotic diseases). To include the best available knowledge for each study site (source and destination areas), we reviewed and compiled information from published literature, non-published information, research or agency reports, and occasional anecdotal accounts. When data was not available for our study sites, we considered published reports of disease affecting Neotropical deer as proxies for potential consequences in each identified hazard (S1). Once the list of potential hazards was completed and shared among the co-authors, together with a comprehensive list of references and resources available for consultation, we considered the baseline knowledge level minimally harmonized and moved to the next step.

#### 2.2.3 Step 3. Risk assessment and prioritization.

Using the same background materials (hazard list, hazard transmission pathway figure, shared literature review S1), we completed an individual risk assessment exercise based on our personal criteria and experience, and assisted by guiding questions (S2). We took eight weeks to perform our individual risk assessments working offline. We used separate assessment tables for each one of the two populations of interest, namely translocated deer and local wildlife (Table 1). Likelihood of exposure was defined as the probability for the population of interest to be exposed to each hazard because of the translocation, and potential consequences of this exposure were scored considering magnitude, severity and life-threatening possibilities. Both parameters were evaluated through a semi-quantitative scale of negligible (1), low (2), medium (3) and high (4). After that, considering risk as the product of Likelihood of Exposure x Consequences [22], the risk value for each hazard was calculated as the numeric product as follows: negligible (0–4), low (5–8), medium (9–12) and high (13–16). Additionally, in the same exercise we stated our confidence in our judgement/scoring, scaling it qualitatively from low, to medium or high.

Our opinions were weighed equally and statistical Mode was used as the summary parameter for all individual assessments (indicating the most frequently occurring value in the dataset). In cases where two values had equal frequencies, we preemptively chose the higher risk value, in alignment with the precautionary principle [22]. Mode was also used to summarise the confidence level assigned, but in this case if two values had equal frequencies, we considered the lower one, assuming also as a precautionary principle that there are still considerable information gaps in our database.

With this, we constructed a risk matrix for each one of the two populations of interest, obtaining risk as the product of Likelihood of exposure Mode x Consequences Mode. For paired risk prioritization (translocated deer and local wildlife), we used a double income table (adapted from Jakob-Hoff et al. [22]), fed with the two previous risk matrices. This allowed qualitative pairing of two different risk levels for the same hazard, and subsequent joint prioritization under the following criteria:

Criterion 1: At least one of the risks (translocated deer or local wildlife) was categorized as moderate or high. The authors met to share and discuss in depth the rationale for subsequent evaluation of suitable risk mitigation measures.

Criterion 2: Risk pairs categorized as low-low. The authors considered it desirable but not a priority to engage in discussions for risk mitigation. Thus, they were discussed in a second priority level, according to interest or concern raised by at least one author.

Criterion 3: Risk pairs categorized as negligible-negligible or low-negligible (and vice versa). These pairs did not warrant further discussion or analysis regarding risk management and were not included in group discussions.

To foster discussion and enhance the outcomes of the exercise, we scheduled a second joint meeting (3hs) in which we shared the most relevant or outstanding concerns about the risk assessment and prioritization process, and achieved consensus on outcomes. During this discussion, seasonal variations in exposure to or consequences of specific hazards were added as input for selection of the best season or time period for translocation during the following DRA step (risk management).

#### 2.2.4 Step 4. Risk management.

Based on the highest paired-ranked risks, we considered hazards matching criteria 1 and 2 (e.g., relevant hazards) for detailed risk assessment in terms of planning mitigation strategies. During two scheduled online meetings (3hs each), we reviewed the hazards transmission pathway to establish critical control points (CCPs) where risks could be minimized or mitigated through specific interventions/measures (Fig 3). To include state of the art techniques used in risk management, we consulted with specific experts in diagnostic tests, parasite eco-epidemiology, and experiences on off-label vaccination or treatment. We discussed different risk management options in terms of their goal/purpose, likely effectiveness and feasibility [22], and achieved a final recommendation of desirable or necessary interventions. When detected, we depicted seasonality related risks for a given hazard in a timetable to allow selecting the lower risk period for animal transportation.

## 3 Results

### 3.1 Hazard identification

We identified 61 hazards potentially associated with translocation of marsh deer from Iberá to Impenetrable, including 32 parasitic: sarcocystosis, neosporosis, toxoplasmosis, coccidiosis, babesiosis, theileriosis, trypanosomiasis (*T. evansi & T. theileri*), horse bot fly, fasciolasis, paramphistomiasis, gastric trematodiasis, parasitic bronchitis, *Gongylonema* infection, *Pygarginema* infection, ascaridiasis, verminous gastroenteritis, capillariasis, black disease, hydatidosis, hard ticks (*Rhipicephalus microplus, Amblyomma triste, Amblyomma* spp., *Dermacentor nitens*), *Lipoptena* infection, fleas, myasis, screw-worm, demodectic mange, chorioptic mange, psoroptic mange and sarcoptic mange; 15 bacterial: leptospirosis, brucellosis, bovine tuberculosis, Johne´s disease (paratuberculosis), enzootic abortion, Q Fever, ehrlichiosis, anaplasmosis (*A. phagocytophilum, A. marginale, A. bovis, A. platys, Candidatus anaplasma boleense*), Shiga toxin-producing *Escherichia coli*, anthrax: 8 viral: infectious bovine rhinotracheitis, malignant catharral fever, foot and mouth disease, vesicular stomatitis, bovine viral diarrhea, enzootic bovine leukosis, bluetongue, epizootic haemorrhagic disease and rabies; 1 prion: chronic wasting disease; 1 fungal: *Aureobasidium* infection; and 3 non-infectious nutritional or management related hazards: capture myopathy, selenium/Vit E deficiency and nutritional deficit. A schematic hazard transmission pathway is presented in Fig 3. No geographical or health-related barriers were identified along the translocation pathway. Site-specific information for each hazard in this study according to best available knowledge can be found in Supporting information 1 (S1). Regarding political boundaries, interprovincial limits to be crossed between source and destination parks presented no specific health regulations for ground transportation of deer.

### 3.2 Risk assessment

Two semi-quantitative risk matrices were constructed using two-way tables after summarising all individual hazard assessments (Tables 2 and 3). After risk assessment discussion rounds, from a total of 61 initially listed hazards, 9 resulted in medium to high qualitative risk for translocated deer (quantitative risk ˃ 8), whereas for local wildlife, medium to high-risk hazards were only 4. Combining those two matrices, results were shared with the team together with the rationale applied to obtain a final Paired Risk Prioritization Tool (PRPT, Table 4). Using the PRPT, we held a fourth online group meeting to discuss appropriate risk mitigation approaches for each hazard under criteria 1 and 2 (10 and 4 hazards respectively, see 2.4), discarding hazards with overall negligible or low-negligible risk scores. No hazard was excluded for the final risk prioritization tool (Table 4). This tool compares risk assigned for the two populations of interest, regarding all 61 listed hazards. Its key feature is matching both risks to assign each hazard a unique prioritization criterion with numeric values. This allows not only to consider all hazards in the same matrix but to prioritize them in order for posterior risk management.

**Table 2:** Risk assessment for hazards affecting “Local wildlife” population of interest.

		LIKELIHOOD of LOCAL WILDLIFE EXPOSURE			
		NEGLIGIBLE (1)	LOW (2)	MEDIUM (3)	HIGH (4)
**CONSEQUENCES for LOCAL WILDLIFE**	**NEGLIGIBLE (1)**	RISK: 1X1 = 1. Malignant catharral fever; Foot & mouth disease; Epizootic hemorrhagic disease; Rabies; Chronic wasting disease; Q fever; *Anaplasma phagocytophilum*; Gastric trematodiasis; *Gongylonema* infection; Black disease; Hydatidosis; *Lipoptena* spp.; *Riphicephalus microplus*; Fleas; Myasis; Screw-worm; Demodectic mange; Psoroptic mange; Capture myopathy; Selenium/Vit E deficiency; Nutritional deficit	RISK: 1X2 = 2. Enzootic bovine leukosis; Babesiosis; *Pygarginema* infection	RISK: 1X3=3 Neosporosis	RISK: 1X4=4. Capillariasis
	**LOW (2)**	none	RISK: 2X2=4. Infectious bovine rhinotracheitis; Vesicular stomatitis; Bovine viral diarrhea; Bluetongue; Paratuberculosis; Enzootic abortion; *Anaplasma bovis; Candidatus anaplasma boleense; E coli*; Toxoplasmosis; *Trypanosoma evansi*; Parasitic bronchitis; Verminous gastroenteritis; *Amblyomma triste; Dermacentor nitens*; Chorioptic mange; Sarcoptic mange; Aureobasidium infection	RISK: 2X3=6. *Anaplasma platys; Anaplasma odocoilei*	RISK: 2X4=8. Leptospirosis; *Anaplasma marginale*; Sarcocystosis; Coccidiosis; *Trypanosoma theileri*; Fasciolasis; Paramphistomiasis; Ascaridiasis; *Amblyomma* spp.
	**MEDIUM (3)**	none	RISK: 3X2=6. Horse Bot Fly	RISK:3X3=9. Bovine Tuberculosis	RISK: 3X4=12. Theileriosis
	**HIGH (4)**	none	RISK; 3X2=6. Anthrax	none	RISK: 4X4=16 Brucellosis; *Ehrlichia chaffeensis.*

Semi-quantitative risk levels for hazards affecting population of interest “local wildlife” in a scenario of marsh deer translocation from Iberá to Impenetrable. Both likelihood of exposure and consequence for each hazard affecting other native wildlife are presented in a qualitative scale but directly associated with numeric scores as follows: negligible = 1, low = 2, medium = 3, high = 4. Quantitative risk level is calculated for each table intersection as the numeric product of likelihood (columns) x consequences (rows). At the same time, numeric risk values are grouped in four categories and depicted with distinctive background colours in the following way: white = negligible risk (numeric values 1-4), light-grey = low risk (numeric values 5-8), medium-grey = medium risk (numeric values 9-12) and dark-grey = high risk (numeric values13-16).

**Table 3:** Risk assessment for “Translocated deer” population of interest.

		LIKELIHOOD of TRANSLOCATED DEER EXPOSURE			
		NEGLIGIBLE (1)	LOW (2)	MEDIUM (3)	HIGH (4)
**CONSEQUENCES for TRANSLOCATED DEER**	**NEGLIGIBLE (1)**	Risk: 1x1 = 1. Malignant catharral fever; Foot and mouth disease; Chronic wasting disease; Enzootic abortion; *Gongylonema* infection; Ascaridiasis; Black disease; Hydatidosis; *Dermacentor nitens*; *Lipoptena* spp; Fleas; Myasis; Demodectic mange; Psoroptic mange; *Aureobasidium* infection	Risk: 1x2 = 2. Q fever; Paramphistomiasis; Gastric trematodiasis; *Pygarginema* infection; Chorioptic mange	Risk: 1x3 = 3. Infectious bovine rhino tracheitis	Risk: 1x4 = 4. Sarcocystosis; Capillariasis
	**LOW (2)**	Risk: 2x1 = 2. Enzootic bovine leukosis; *Anaplasma phagocytophilum*; Sarcoptic mange	Risk: 2x2 = 4. Vesicular estomatitis; Paratuberculosis; Anaplasma (other than phagocytophilum); *E coli*.; Neosporosis; Toxoplasmosis; Babesiosis.; Trypanosoma theileri; Horse Bot Fly; *Amblyomma triste*; Selenium/Vit E deficiency	Risk: 2x3 = 6. Bovine viral diarrhea; *Ehrlichia chaffeensis*; Screw-worm	Risk: 2x4 = 8. Leptospirosis; Coccidiosis; *Trypanosoma evansi; Amblyomma* spp.
	**MEDIUM (3)**	none	Risk: 3x2 = 6. Bluetongue	Risk: 3x3 = 9. Theileriosis; Parasitic bronchitis; *Riphicephalus microplus*; Verminous gastroenteritis; Nutritional deficit	Risk: 3x4 = 12. Fasciolasis
	**HIGH (4)**	Risk: 4x1 = 4. Epizootic hemorrhagic disease	Risk: 4x2 = 8. Anthrax	none	Risk: 4x4 = 16. Rabies; Brucellosis; Bovine tuberculosis; Capture myopathy

Semi-quantitative risk levels for hazards affecting population of interest “translocated deer” in a scenario of translocation from Iberá to Impenetrable. Both likelihood of exposure and consequences for each hazard affecting translocated deer are presented in a qualitative scale but directly associated with numeric values as follows: negligible = 1, low = 2, medium = 3, high = 4. Quantitative risk level is calculated for each table intersection as the numeric product of likelihood of exposure (columns) x consequences (rows). At the same time, numeric risk values are grouped in four categories and depicted with distinctive background colours in the following way: white = negligible (1-4), light-grey = low (5-8), medium-grey = medium (9-12) and dark-grey = high (13-16).

**Table 4:** Risk prioritization of hazards for populations of interest and zoonotic diseases.

Hazard	Populations of interest		Prioritization Criteria °	Confidence (Statistical Mode)	Zoonotic Potential
	Translocated Deer Risk*	Wildlife Risk*			
**Rabies** (Rabies virus)	16 (high)	1 (negligible)	1	medium	yes
**Brucellosis** (*Brucella* spp.)	16 (high)	16 (high)	1	medium	yes
**Bovine Tuberculosis** (*Mycobacterium bovis*)	16 (high)	9 (medium)	1	medium	yes
**Human Monocytic Ehrlichiosis** (*Ehrlichia chaffeensis*)	6 (low)	16 (high)	1	medium	yes
**Theileriosis** (*Theileria cervi, T. equi*)	9 (medium)	12 (medium)	1	medium	no
**Fasciolasis** (*Fasciola hepatica*)	12 (medium)	8 (low)	1	medium	yes
**Verminous Gastroenteritis** (*Strongyloides* spp*, Trichostrongylus* spp*., Haemonchus* spp*., Ostertagia* spp*., Cooperia* spp*., Oesophagostomum* spp*., Trichuris* spp*.*)	9 (medium)	4 (negligible)	1	medium	no
**Hard Ticks** *Rhipicephalus microplus*	9 (medium)	4 (negligible)	1	medium	yes
**Nutritional deficit**	9 (medium)	1 (negligible)	1	low	no
**Capture myopathy**	16 (high)	1 (negligible)	1	medium	no
**Leptospirosis** (*Leptospira* spp.)	8 (low)	8 (low)	2	medium	yes
**Anthrax** (*Bacillus anthracis*)	8 (low)	8 (low)	2	medium	yes
**Coccidiosis** (*Eimeria* spp*., Isospora* spp*.*)	8 (low)	8 (low)	2	medium	no
**Hard ticks** (*Amblyomma* spp*.)*	8 (low)	8 (low)	2	low	yes
**Infectious Bovine Rhinotracheitis** (BoHV: 1.2a/1.2b/4/5)	3 (negligible)	4 (negligible)	3	low	no
**Malignant Catarrhal Fever** (AlHV-1/OvHV-2)	1 (negligible)	1 (negligible)	3	low	no
**Foot and mouth Disease** (Foot and mouth disease virus)	1 (negligible)	1 (negligible)	3	medium	yes (low)
**Vesicular Stomatitis** (Vesicular stomatitis virus)	4 (negligible)	4 (negligible)	3	low	yes
**Bovine Viral Diarrhea** (Bovine viral diarrhea virus)	6 (low)	4 (negligible)	3	low	no
**Enzootic Bovine Leukosis** (Bovine leukaemia virus -BLV)	2 (negligible)	2 (negligible)	3	low	no
**Bluetongue** (Bluetongue virus)	6 (low)	4 (negligible)	3	medium	no
**Epizootic Hemorrhagic Disease** (Epizootic hemorrhagic disease virus)	4 (negligible)	1 (negligible)	3	low	no
**Chronic Wasting Disease** (CWD)	1 (negligible)	1 (negligible)	3	low	yes (low)
**Johne’s disease-Paratuberculosis** (*M. avium paratuberculosis*)	4 (negligible)	4 (negligible)	3	medium	no
**Enzootic Abortion** (*Chlamydia abortus*)	1 (negligible)	4 (negligible)	3	low	yes
**Q Fever** (*Coxiella burnetti*)	2 (negligible)	1 (negligible)	3	low	yes
**Anaplasmosis** (*Anaplasma phagocytophilum*)	2 (negligible)	1 (negligible)	3	low	yes
**Anaplasmosis** (*A. marginale*)	4 (negligible)	8 (low)	3	medium	no
**Anaplasmosis** (*A. bovis*)	4 (negligible)	4 (negligible)	3	low	no
**Anaplasmosis** (*A. platys*)	4 (negligible)	6 (low)	3	low	no
**Anaplasmosis** (*A. odocoilei*)	4 (negligible)	6 (low)	3	low	no
**Anaplasmosis** (*Candidatus anaplasma boleense*)	4 (negligible)	4 (negligible)	3	low	no
**Shiga toxin producing** ***Escherichia coli***	4 (negligible)	4 (negligible)	3	low	yes
**Sarcocystosis** (*Sarcocystis* spp*.*)	4 (negligible)	8 (low)	3	medium	yes
***Neosporosis*** (*Neospora* spp.)	4 (negligible)	3 (negligible)	3	medium	no
**Toxoplasmosis** (*Toxoplasma gondii)*	4 (negligible)	4 (negligible)	3	medium	yes
**Babesiosis** (*Babesia bovis y B. bigemina*)	4 (negligible)	2 (negligible)	3	low	no
**Trypanosomiasis** (*Trypanosoma evansi*)	8 (low)	4 (negligible)	3	medium	no
**Trypanosomiasis** (*T. theileri*)	4 (negligible)	8 (low)	3	medium	no
**Horse Bot Fly** (*Gasterophilus intestinalis* & *G. nasalis*)	4 (negligible)	6 (low)	3	low	no
**Paramphistomiasis** (*Paramphistomum cervi, P. liorchis*)	2 (negligible)	8 (low)	3	medium	no
**Gastric trematodiasis** (*Balanorchis anastrophus*)	2 (negligible)	1 (negligible)	3	low	no
**Parasitic bronchitis** (*Dictyocaulus* spp.)	3 (negligible)	4 (negligible)	3	low	no
**Gongylonema infection** (*Gongylonema* spp*.*)	1 (negligible)	1 (negligible)	3	low	yes
**Pygarginema infection** (*Pygarginema verrucosa*)	2 (negligible)	2 (negligible)	3	low	no
**Ascaridiasis** (*Ascaris* spp.)	1 (negligible)	8 (low)	3	low	yes
**Capillariasis** (*Capillaria* spp.)	4 (low)	4 (negligible)	3	low	no
**Black disease** (*Thysanosoma actinoides*)	1 (negligible)	1 (negligible)	3	low	no
**Hydatidosis** (*Echinococcus granulosus*)	1 (negligible)	1 (negligible)	3	low	yes
**Hard Ticks** (*Amblyomma triste*)	4 (negligible)	4 (negligible)	3	medium	yes
**Hard ticks** (*Dermacentor nitens*)	1 (negligible)	2 (negligible)	3	low	yes
***Hematophagous louse-fly*** **(***Lipoptena* spp.)	1 (negligible)	1 (negligible)	3	low	no
**Fleas** (*Ctenocephalides felis*)	1 (negligible)	1 (negligible)	3	low	yes
**Myasis** (*Dermatobia hominis*)	1 (negligible)	1 (negligible)	3	low	yes
**Screw-worm** (*Cochliomyia hominivorax*)	3 (negligible)	1 (negligible)	3	low	yes
**Demodectic mange** (*Demodex* spp.)	1 (negligible)	1 (negligible)	3	low	no
**Chorioptic mange** (*Chorioptes* spp.)	1 (negligible)	2 (negligible)	3	low	no
**Psoroptic mange** (*Psoroptes* spp.)	1 (negligible)	1 (negligible)	3	low	no
**Sarcoptic mange** (*Sarcoptes* spp*.*)	2 (negligible)	4 (negligible)	3	low	yes
**Aureobasidium infection** (*Aureobasidium pullulans*)	1 (negligible)	1 (negligible)	3	low	yes
**Selenium/Vit E deficiency**	4 (negligible)	1 (negligible)	3	low	no

* Quantitative values and associated qualitative value: 1–4 (negligible), 5–8 (low), 9–12 (medium), 13–16 (high), for more details see footnotes in Tables 2–3.

° Prioritization criteria: 1-at least one of the risks (translocated deer or local wildlife) categorized as moderate or high, 2-risk pairs categorized as low-low, 3-risk pairs categorized as negligible-negligible or low-negligible (and vice versa).

Paired Risk Prioritization Tool for all populations of interest, including whether the disease is zoonotic which implies additional risk for humans. The Confidence column represents the statistical Mode of each author’s confidence in their own judgement/scoring during risk assessment for each hazard, scaling qualitatively from low, to medium or high.

In terms of confidence for each individual assessment, among 61 listed hazards none was rated high (0%), 23 were rated medium (38%), and coinciding with the context of high uncertainty and information gaps, 38 were rated as low (62%). Despite that, among the 14 hazards meeting criteria 1 and 2, 86% were rated as medium and only 14% rated low confidence.

### 3.3 Risk management

Mitigation for hazards with relevant associated risk was discussed and grouped around strategic Critical Control Points (CCPs). We identified six CCPs appropriate for mitigation interventions throughout the hazard transmission pathway (Fig 3). CCP1 (On-Site Quarantine) was focused on in situ pre-translocation health-checks and medical prophylaxis (vaccination, deworming, etc.) to reduce risk of translocating pathogen carriers. CCP2 (Ground Transport) involved best practices specific for wildlife including transport cleaning, disinfecting and ensuring animal welfare (health and mental conditions). For CCP3 (Pre-Release Pen), the focus was on providing a second chance to control and minimise the involuntary transport of pathogens. Although a second strict quarantine at the destination site could have been considered, the substantial complications associated with post transportation stress, animal management and wild-caught free-ranging deer (such as stress, decreased food intake, and trauma) led us to opt for suggesting a large holding pen at the destination site. This pre-release holding pen would facilitate the adaptation of transferred individuals to the local meteorological conditions and environment, while allowing routine monitoring and visual assessment of individuals. Medical practices would be limited to non- invasive, except for emergency care needs. The supply of drinking water and food would be a key factor at this stage, with a focus on the gradual natural dietary transition to locally available foods. CCPs 4–6 aimed at monitoring deer survival as well as the early detection and diagnosis of potential disease issues related to contact between deer and local animals. Risk management options for each CCP, their assessment and final recommendation as necessary or desirable, are presented in Table 5. Biosecurity precautions to minimise staff and animal exposure to zoonotic/anthroponotic diseases were considered necessary throughout the entire translocation procedure. It was agreed that protocols should include the use of disinfectant foot baths, appropriate clothing and personal protective elements, while minimising physical contact with the animals. Although appropriate prophylactic vaccination of the quarantine staff exceeded the scope of this project, it should be considered following physician guidance.

**Table 5:** Critical control points (CCPs), associated risk mitigation options and final recommendation.

CCP1: Strict quarantine and health check	Recommendation
Strict quarantine facilities and protocols	Necessary
Skilled staff (veterinarians, technicians, keepers) for chemical restraint and quarantine holding	Necessary
Anthrax vaccination	Desirable
Brucellosis, Rabies & Leptospirosis vaccination	Desirable
Ectoparasitic treatment	Necessary
Verminous gastroenteritis targeted treatment	Necessary
*Ehrlichia chaffeensis* (paired PCR)	Necessary
*Theileria cervi* (paired PCR)	Necessary
Foot and mouth negative serology	Necessary
Leptospirosis negative serology	Necessary
Brucellosis negative serology	Necessary
SARS-Cov2 (negative PCR)	Necessary
Negative SENASA health check ^*^	Necessary
Personal protective elements (PPE) & biosafety protocols for staff	Necessary
Animal welfare considerations for captive wildlife	Necessary
**CCP2: Ground transportation**	
Transport hygiene & disinfection	Necessary
Individual or low animal numbers during transportation	Necessary
Trailer especially adapted for wild-deer	Necessary
Long action tranquilizers (e.g., zuclopentixol)	Desirable
Skilled staff (veterinarians, technicians, keepers) for transportation	Necessary
Personal protective elements (PPE) & biosafety protocols for staff	Necessary
**CCP3: Pre-release holding pen**	
Pre-release holding facilities	Necessary
Safe water & food for nutritional transition	Necessary
Animal welfare considerations for captive wildlife	Necessary
Personal protective elements (PPE) & biosafety protocols for staff	Necessary
**CCP4: Livestock contact**	
Gathering baseline information on neighboring livestock health	Necessary
Systematic health survey on neighboring livestock	Desirable
Preventing or regulating the entry of neighboring domestic livestock into Impenetrable	Necessary
Erradicate/control feral livestock inside Impenetrable	Necessary
Establish-implement an articulated neighboring livestock management plan for Impenetrable	Desirable
Personal protective elements (PPE) & biosafety protocols for staff	Necessary
**CCP5: Wildlife interactions (native & exotic)**	
Gathering baseline information on wildlife health	Necessary
Wildlife systematic health monitoring	Desirable
Personal protective elements (PPE) & biosafety protocols for staff	Necessary
Implementation/enforcement of alien/invasive species	Necessary
**CCP 6: Translocated-deer monitoring**	
Non-invasive health monitoring	Necessary
Mortality monitoring (including full necropsies)	Necessary
Habitat use monitoring	Necessary
Personal protective elements (PPE) & biosafety protocols for staff	Necessary

* Argentinean animal health officers indicate testing for Foot and mouth disease, Enzootic bovine leukosis, IBR, BVD, Q Fever, Bluetongue, Leptospirosis, Brucellosis and Enzootic abortion.

Critical control points (CCPs) identified for the scenario of translocation of marsh deer from Iberá to Impenetrable, associated risk mitigation options, and final recommendation as absolutely necessary to ensure acceptable risk, or desirable if available to voluntarily reduce risk.

At this stage, differential consideration was given to SARS-CoV-2 risk mitigation, given recorded exposure in cervids in North America [55–57]. Even though for the moment there is no evidence of viral circulation in neotropical cervids, since the COVID-19 pandemic the government of Argentina recommends testing and notification of SARS-CoV-2 when transporting wildlife. Thus, this hazard was included in the risk mitigation actions.

Risk seasonality was addressed during risk mitigation discussion rounds. Acknowledging risk is never zero, we identified a preferred seasonal time period for transportation that would minimise associated risk. Two main factors identified were the seasonality of previously recorded mass mortality events [45] and the life cycle of *Fasciola hepatica* [58]*.* Additional risks included breeding season, presence of antlers and environmental temperature. These factors and the months affected by them are summarized in Table 6, where the period with lowest risk for transportation was identified as March to May (months with more green or yellow shade when considering the five rows).

**Table 6:** Seasonal variations in hazard-related risks for marsh deer (*Blastocerus dichotomus*) in the proposed translocation scenario in Argentina.

Seasonal Hazard-related Risk	Jan	Feb	Mar	Apr	May	Jun	Jul	Aug	Sep	Oct	Nov	Dec
**Reproductive Season** [59–61]				fawn births					mating			
**Antler presence (males)** [43]												
***Fasciola Hepatica°***						high loads in definitive host				infective forms		
**Temperature°**												
**Mass Mortality events [46]**												

°This study.

**Seasonal variations in hazard-related risks for marsh deer**. This table outlines seasonal variations in hazard-related risks, including reproductive patterns, antler presence in males, *Fasciola hepatica* infections, temperature variations, and recorded mass mortality events. It shows how these factors fluctuate throughout the year. Cells shaded in green indicate low risk, yellow indicates moderate risk and orange high risk. Specific details on fawn births, mating periods, antler presence, and infection loads, are detailed in the text.

## 4 Discussion

The period 2020–2030 has been proposed as the United Nations Decade on Ecosystem Restoration [62], to prevent, halt and reverse the degradation of ecosystems worldwide. Wildlife translocations are part of the ecological restoration toolkit, but unlike other methods they carry inherent risks of unwanted effects, such as inadvertent pathogen introduction. Mitigation strategies like DRA are pivotal for the success of restoration initiatives involving wildlife translocations. Using a case study, we show how, even in low resource settings, integrating DRA can enhance planning and outcomes of conservation translocations, increasing certainty within a highly uncertain process.

We based the marsh deer translocation scenario on an existing project in Argentina to test the applicability of DRA in the local context. Justification of the existing translocation project exceeds this research’s objectives, was not discussed by authors, and is in no way endorsed by authors by applying DRA to this case.

The fact that 61 potential hazards were identified underscores the complexity of the process, magnified by data gaps and deficiencies, and highlights our meticulous approach at multiple scales to ensure thorough consideration of multiple risk factors. The use of semi-quantitative risk matrices and the consolidation of individual assessments enabled the identification of nine priority hazards with medium to high risk for the translocated deer, and four for local wildlife. Then, approaching risk prioritization collaboratively and from diverse disciplinary perspectives ensured that the most critical hazards were effectively addressed. This was followed by reviewing and identifying pathways to mitigate selected risks, thus ensuring an adaptive and evidence-based approach to translocation. While potentially overcautious, we felt strongly about minimizing risks to the extent possible.

In our case study, the identification of hazards resulted in a large number of diseases listed, doubling other published DRAs such as Verant et al. [24], and included potential impacts on the health of wildlife, domestic animals, and humans. Three main reasons account for listing so many diseases: (1) authors intentionally included a wide variety of profiles and experiences in wildlife health, domestic animal health, and One Health, enabling the inclusion of diverse and often neglected diseases affecting health and well-being in various ways; (2) including local wildlife as a population of interest expanded the spectrum of hosts and associated diseases; (3) given the lack of empirical disease knowledge for Impenetrable, the precautionary principle suggested not underestimating any disease risk, leading us to list even low-risk hazards. Nevertheless, after careful evaluation, only 14 hazards were further addressed, having obtained medium to high-risk scores.

During group sessions, we analyzed and discussed rationale underlying individual assessments and aimed for group consensus. Most of the prioritized diseases are common in domestic animals. Therefore, times and spaces in which overlap between wildlife and livestock may occur, before/at source, during transportation and after/at destination, were identified as critical control points for transmission. This was important because certain diseases considered controllable when restricted to livestock species may become almost impossible to eradicate once they spread to free-ranging wildlife (e.g., chronic wasting disease and bovine tuberculosis) [63]. On the other hand, endemic livestock pathogens in the destination area may put healthy but naïve translocated animals at risk (e.g., infection with endemic *Theileria* spp. and parasitism by *Elaeophora elaphi* in naïve red deer imported from Germany to Spain) [64].

When assessing risk for local wildlife at Impenetrable, discussions centred on: (a) diseases that according to the authors knowledge are not present in/reported for the area (e.g., *E. chaffeensis*), (b) diseases that affect/impact a large number of species (e.g., tuberculosis, anthrax, brucellosis, leptospirosis), and (c) diseases carried by translocated deer that could increase wildlife morbidity/mortality rates and/or decrease fertility beyond expected levels (e.g., brucellosis, theileriosis, tuberculosis).

Regarding risks for translocated deer, translocation-induced stress was identified and considered a predictable factor [65], that even when incorporated in translocation planning (in terms of mitigation goals) would undoubtedly increase vulnerability to listed hazards. According to Dickens et al. [65] two main stresses can be differentiated: an initial adaptive acute stress and a posterior maladaptive chronic stress. The first one encompasses adaptive physiological and behavioural responses to cope with a stressor focusing on immediate survival needs. Such a response is generally beneficial to the individual, but in some cases can lead to major complications. As an example, capture myopathy is a complex syndrome involving muscle injuries as the result of an extreme fight-or-flight response associated with the acute stress. It has been identified as a major source of mortality during capture of deer and other species [66], but can also produce long-term effects, significantly affecting overall body condition [67] and ability to escape predation following release [68]. A relevant risk score was assigned to capture myopathy in this specific case study, considering wild deer captures, repeated sampling procedures and ground transportation to an unknown environment. The second, chronic stress, can occur if the stressor persists or multiple acute stressors initiate consecutively [69]. Translocation has significant potential to induce chronic stress, as the process involves numerous acute stressors cascading acute stress responses as well as long-term responses. Even routine translocations typically include capture and handling, captivity or prolonged restraint, transport, and release into an unfamiliar environment. Under that scenario, the original short-term physiological and/or behavioural changes crucial for coping with acute stressors no longer aid in survival and instead become detrimental [70]. Reproductive physiology and behaviour and immune system maintenance (among others) are unnecessary in the context of immediate survival and the prolonged acute stress response acts to temporarily arrest these systems, increasing vulnerability to infectious/parasitic or non-infectious hazards [65].

Beyond translocation-induced chronic stress, the transportation of deer carrying pathogens (e.g., *E. chaffeensis*, *Fasciola* spp.) or disease vectors (e.g.*,* ticks *Rhipicephalus microplus, Amblyomma* spp.) was considered highly undesirable due to increased chances of triggering illness and/or disease spreading. At the same time, diseases undermining body condition (e.g., verminous gastroenteritis, parasitic bronchitis, fasciolasis) could lead to lesions or health problems with consequences above those expected for healthy, undisturbed individuals. The same reasoning applied for diseases compromising fertility (e.g., brucellosis), undermining the establishment of a new self-sustaining population under the context of translocation-induced chronic stress with a potentially suppressed immune system and neglected reproductive physiology.

In this DRA, sixty six percent (8/14) of the prioritized diseases have zoonotic potential as one factor associated with higher risk. Notwithstanding, we assigned high potential consequences only to *E. chaffeensis,* the pathogen causing Human Monocytic Ehrlichiosis, a disease not yet reported in humans in Argentina. This pathogen has been detected infecting marsh deer and in *R. microplus* ticks in Iberá wetlands [71]. Moreover, *E. chaffeensis* has also been found in *A. parvum* ticks collected from domestic ruminants and canids in northern Argentina [72]. Nevertheless, little is known about other mammalian hosts and vectors involved in its transmission cycle, and it is unknown whether it is present at the destination area. Thus, in the current scenario, following precautionary principle and unless new evidence becomes available, it was assumed that *E. chaffeensis* is absent in Impenetrable. Therefore, potential disease translocation to a naïve environment could precipitate disease emergence and spread, especially considering expected chronic stress-related immunosuppression in carrier deer.

The seasonality of risk, including factors such as observed mass mortality events and the life cycle of *Fasciola hepatica*, was addressed during risk mitigation discussions. We identified a low-risk period for transportation, from March to May, demonstrating that strategic planning sensitive to seasonal variations is possible. Additionally, the inclusion of emerging risks such as SARS-CoV-2, despite no evidence of the virus in Neotropical deer, reflects the plan’s adaptability to changing epidemiological contexts and compliance with governmental guidelines.

At the onset of the DRA exercise, it was noted that beyond pathogens some other factors like environmental and/or climatic issues may act as potential disease triggers and must thus be considered. For example, physical condition and immunocompetence may deteriorate due to physiological and behavioural responses to increased energetic demands when exploring new territories [73,74], and chronic malnutrition associated with utilisation of marginal habitat has been associated with increased deer mortality [75]. In addition, the transition from high humidity weather at Iberá to the dry forest of Impenetrable, together with changes in micronutrients and drinking water availability, may further immunosuppress or predispose translocated animals to disease [76]. In this case study, the deer would not only undergo significant stress inherent to the translocation process itself, but could be subjected to further stress by being placed in a potentially unsuitable environment. It is uncertain whether the Impenetrable habitat provides the resources or conditions necessary for optimal survival and it is hypothesized that it may not meet the specific dietary, social, and shelter needs of marsh deer. These restrictions could increase deer vulnerability to disease and predation. For these reasons it would be imperative to monitor and manage these risks carefully, complementing DRA with broad ecological and environmental suitability analyses, to ensure the long-term success and health of the translocated population.

We identified six CCPs for mitigation interventions along the hazard transmission pathway (Fig 3). However, starting from CCP1, selecting deer negative to *E. chaffeensis* may prove challenging since negative results cannot completely rule out infection, even using highly sensitive tests like Polymerase Chain Reaction (PCR) [77]. Based on that particularly relevant red-flag associated with this hazard, we considered that under the current scenario, the overall disease risk for the translocation would be unacceptably high from the One Health perspective. While serological techniques could be proposed as a complementary diagnostic method, they have limitations since they only detect exposure to the agent (indicated by circulating antibodies), and the duration of *E. chaffeensis* antibodies in marsh deer remains unknown. Since the agent is present in the source area, we would expect a certain exposure level of exposure in the source deer population, even after a negative PCR. Unfortunately, in Argentina serology for detecting antibodies against *Ehrlichia spp* is only available in the form of point-of-care rapid tests for *Ehrlichia canis*, and serology for *E. chaffeensis* is not available for neither human nor deer. Thus, diagnostics would require shipping samples overseas, which is impractical and time consuming for wildlife due to regulations like the Convention on International Trade in Endangered Species of Wild Fauna and Flora (CITES), and/or validating a high sensitivity and specificity technique in the country, which would require additional time and financial resources. This would add extra difficulties in interpreting non-specific diagnostic results because of potential cross-reactivity. Considering this limitation to addressing the highest risk of spreading a zoonotic hazard, we suggested adding a second screening strategy oriented to increase efforts in detecting the agent at the destination area. If the presence and circulation of *E. chaffeensis* in Impenetrable is sufficiently confirmed, then and only then, mitigation options aimed at reducing the risk of transporting positives or carriers to the lowest level, would be considered acceptable. That said, implementation of on-site quarantine (CCP1) and prophylactic measures such as vaccination and targeted deworming, along with specific stress reducing transport practices for deer (CCP2) were considered fundamental and thus necessary mitigation strategies. The establishment of a pre-release enclosure at the destination site (CCP3) emerged as a practical option to minimize stress and facilitate deer adaptation to the new environment (lowering chronic-stress triggers), while enabling monitoring and health assessment of translocated individuals.

Conservation translocation projects are by definition open-ended, which increases perceived and real uncertainty [78] and adds complexity to both defining and monitoring their success [79,80]. Without quantitative means to monitor and evaluate progress, conservation translocation projects will lack the tools to oversee and learn from their execution [81,82]. These requirements should not be taken lightly, since many monitoring strategies also provide hazard mitigation and feedback to the translocation model through their results [83]. Considering significant effects of conservation translocation on the ecosystem may take several years to emerge, substantial funding and technical resources may be needed to cover this timeframe [83,84]. Different monitoring frameworks specific for measuring conservation translocation progress have been developed [85,86] with the aim to provide a more quantitative evaluation. Among them, remote sensing has been used extensively for monitoring translocated animal movement and distribution, changes in habitat area, habitat degradation, but also temporal or spatial trends in pressures and threats [87]. In this case study and with more modest aims, CCPs 4–6 were proposed in order to establish a solid post-release monitoring strategy, involving not only deer survival but disease circulation at the interface between deer, other wildlife and domestic animals. Thus, the full risk mitigation options proposed for this study case included 39 options of which all but 6 were considered necessary. This is strongly related not only to the risk values in the assessment, but also to the many uncertainties and grey areas associated with wildlife health knowledge in our region. All considered, financial, technical and logistical costs associated with proper mitigation may be high, which in terms of feasibility could represent a major challenge for proposals like this to be implemented responsibly under the current scenario.

Overall, the objectives of this exercise were achieved. Even while encountering challenges in implementing the DRA methodology, the process promoted valuable consensus-building among participants. The diversity of disciplines and sectors involved posed minor difficulties such as different interpretation of technical terms and varying worldviews, which were resolved by facilitated communication. Likewise, the stepwise approach guided by a facilitator was crucial for clarifying and integrating knowledge, ensuring each phase was comprehensively executed. Another challenge was maintaining focus on disease risk assessment rather than integrating broader conservation aspects and implications (e.g., conflicting interests, social factors), requiring careful facilitation to keep the group aligned with the DRA’s specific mandate. However, the simplified format of this DRA, based solely on the expertise of our core group and without broader stakeholder engagement or validation, represents a limitation. The lack of input from local communities and other stakeholders may have excluded alternative perspectives, social and ecological trade-offs, and varying levels of risk tolerance (i.e., acceptable risk). A wider consultation could have enriched the assessment, offering a more comprehensive and balanced approach to decision-making.

The inclusion of two distinct populations of interest (deer and non-deer native wildlife) initially added complexity to the assessment. While human health risks were also considered, the decision not to include humans as a third population of interest allowed for examination of zoonotic diseases and their potential consequences across multiple species rather than solely from a human-centric perspective. Still, this approach resulted in two complementary risk matrices that effectively captured high-consequence zoonotic pathogens, highlighting their prioritization for risk mitigation strategies. Additionally, the site-agnostic approach, which focused on populations rather than specific locations, provided greater flexibility and applicability. By emphasizing the species of concern, this approach ensured the assessment’s relevance across different ecological contexts, while maintaining its broad utility for various geographical areas.

Acknowledging uncertainty as inherent in transparent, evidence-based DRAs, our approach identified several limitations during several phases, including the problem description phase. The lack of comprehensive information on disease presence and impacts across different sites and wildlife species increased uncertainty in hazard and risk assessments. The precautionary principle guided the listing of many diseases as hazards, although subsequent detailed assessment significantly reduced the number of medium to high-risk hazards. Our confidence in our assessments for highest risk hazards (under criteria 1 and 2) was predominantly moderate (86%), relying on more robust data for diseases in domestic animals compared to wildlife, and necessitating extrapolation based on professional judgement. Should a project like the one presented here be considered, future research addressing these knowledge gaps before decision-making processes would be crucial to ensuring the safety of the assumptions.

While our study successfully engaged a wide range of experts through online meetings, budget and time constraints remain limiting factors for participatory tools like DRA. Within the chosen approach, cost-effective alternatives such as online platforms for data sharing, coupled with asynchronous work, facilitated diverse participation but also posed challenges. For example, having to work with a reduced number of participants, acknowledging internet connectivity issues and ensuring equitable access. These trade-offs highlight opportunities for future enhancements in robustness and validation through securing resources to allow for in-person interactions, particularly in dealing with complex scenarios requiring broad stakeholder engagement and iterative validation processes.

## 5 Conclusions

Even with acknowledged limitations and uncertainties, this is the first formal report of DRA for conservation translocations between protected areas in Argentina, applied in and adapted to a local context of limited resources and considerable information gaps. Besides recognizing the need for increasing baseline information and resources allocated for these initiatives, we encourage researchers and wildlife officers not to wait for ideal circumstances or enabling environments, but to acknowledge up front the need for adaptation of DRA principles to the local context, seeking advice or assistance as needed (i.e.,: IUCN Rewilding Working Group and Conservation Planning Specialist Group). Even small contributions at a local or regional level will undoubtedly increase the quality of future disease risk assessments in similar contexts.

For the specific case study of marsh deer translocation, the DRA identified *E. chaffeensis* at the source but not at the destination site as the paramount hazard, leading to unacceptable disease risk according to the best available knowledge. Other prioritized hazards, including infectious, non-infectious diseases, chronic stress induced by the translocation, and seasonal considerations were also identified and considered relevant, as they could compromise the health and survival of the translocated animals and/or local wildlife. Those hazards (other than *E. chaffeensis*) were considered plausible for risk mitigation through strategic planning. Thus, efforts to confirm changes in the status of *E. chaffeensis* at the destination area are needed should this project be considered again in the future.

The complexity of conservation translocations is underscored by the fact that disease risk is only one of many factors that must be carefully and transparently evaluated. In this context, DRA serves as a critical tool, offering a structured approach to identifying these risks and mitigating them if possible. However, these results must be incorporated into a broader ecological assessment that considers management goals and other essential factors exceeding our analysis, such as historical species distribution, threats, habitat suitability, local communities’ perception and involvement, native species interactions, and other potential environmental impacts.

## Supporting information

S1 Hazard presence, relevance and references for each study area. Relevant information for hazards presence and relevance on *B. dichotomus*, wildlife (mammals), domestic mammals, and humans, according to source and destination areas.
(XLSX)

S2 Guiding questions for individual risk assessment. These questions will guide you for quantitatively assessing both likelihood of exposure and consequences assigned to each hazard in relation to populations of interest.
(DOCX)

## References

[1] Watson EM, Evans T, Watson JEM, Venter O, Williams B, Tulloch A, et al. The exceptional value of intact forest ecosystems. Nat Ecol Evol [Internet]. 2018 [cited 2024 Apr 15];2(4):15. doi: 10.1038/s41559-018-0490-x 29483681

[2] Foley JA, Defries R, Asner GP, Barford C, Bonan G, Carpenter SR, et al. Global consequences of land use. Science. 2005;309(5734):570–4. doi: 10.1126/science.1111772 16040698

[3] Myers SS, Gaffikin L, Golden CD, Ostfeld RS, Redford KH, Ricketts TH, et al. Human health impacts of ecosystem alteration. Proc Natl Acad Sci U S A. 2013;110(47):18753–60. doi: 10.1073/pnas.1218656110 24218556 PMC3839693

[4] Langridge J, Sordello R, Reyjol Y. Outcomes of wildlife translocations in protected areas: what is the type and extent of existing evidence? A systematic map protocol. Environ Evid. 2020;9(1).

[5] Mitchell RJ. The amplification of plant disease risk through ecological restoration. Restor Ecol [Internet]. 2023 Jul 1 [cited 2024 Apr 15];31(5):e13937. Available from: https://onlinelibrary.wiley.com/doi/full/10.1111/rec.13937

[6] Berger-Tal O, Blumstein DT, Swaisgood RR. Conservation translocations: a review of common difficulties and promising directions. Anim Conserv [Internet]. 2020 [cited 2024 Apr 14];23:121–31. Available from: https://zslpublications.onlinelibrary.wiley.com/doi/10.1111/acv.12534

[7] Buckley MC, Crone EE. Negative off-site impacts of ecological restoration: understanding and addressing the conflict. Conserv Biol [Internet]. 2008 [cited 2024 Apr 14];22(5):1118–24. Available from: https://conbio.onlinelibrary.wiley.com/doi/10.1111/j.1523-1739.2008.01027.x 18759779 10.1111/j.1523-1739.2008.01027.x

[8] Seddon PJ, Griffiths CJ, Soorae PS, Armstrong DP. Reversing defaunation: Restoring species in a changing world. Science (1979) [Internet]. 2014 Jul 25 [cited 2024 Apr 15];345(6195):406–12. Available from: https://www.science.org/doi/10.1126/science.1251818 25061203 10.1126/science.1251818

[9] Massei G, Quy RJ, Gurney J, Cowan DP. Can translocations be used to mitigate human - wildlife conflicts?. Wildl Res. 2010;37(5):428. doi: 10.1071/wr08179

[10] Brichieri-Colombi TA, Moehrenschlager A. Alignment of threat, effort, and perceived success in North American conservation translocations. Conserv Biol. 2016;30(6):1159–72. doi: 10.1111/cobi.12743 27119768

[11] Griffith B, Scott JM, Carpenter JW, Reed C. Translocation as a species conservation tool: status and strategy. Science. 1989;245(4917):477–80. doi: 10.1126/science.245.4917.477 17750257

[12] Nogués-Bravo D, Simberloff D, Rahbek C, Sanders NJ. Rewilding is the new Pandora’s box in conservation. Curr Biol. 2016;26(3):R87–91. doi: 10.1016/j.cub.2015.12.044 26859272

[13] Telfer S, Lambin X, Birtles R, Beldomenico P, Burthe S, Paterson S, et al. Species interactions in a parasite community drive infection risk in a wildlife population. Science [Internet]. 2010 [cited 2024 Apr 15];330(6001):243–6. Available from: www.sciencemag.org/cgi/content/full/330/6001/243/DC1 20929776 10.1126/science.1190333 PMC3033556

[14] Northover A, Lymbery A, Wayne A, Godfrey S, Thompson R. The hidden consequences of altering host-parasite relationships during fauna translocations. Biol Conserv. 2018;220:140–8.

[15] Gakuya F, Kock R, Lekolool I, Mihok S. Trypanosomiasis in introduced Southern White Rhinoceros (Ceratotherium simum simum) gifts to ex situ habitat in Aitong, Kenya. Available from: http://meridian.allenpress.com/jwd/article-pdf/doi/10.7589/JWD-D-24-00026/3421469/10.7589_jwd-d-24-00026.pdf 10.7589/JWD-D-24-00026 39166333

[16] Kock RA, Woodford MH, Rossiter PB. Disease risks associated with the translocation of wildlife. Rev Sci Tech. 2010;29(2):329–50. doi: 10.20506/rst.29.2.1980 20919586

[17] Martel A, Blooi M, Adriaensen C, Van Rooij P, Beukema W, Fisher M. Recent introduction of a chytrid fungus endangers Western Palearctic salamanders. Science. 2014;346(6209):630–1. 25359973 10.1126/science.1258268 PMC5769814

[18] Sainsbury A, Yu-Mei R, Ågren E, Vaughan-Higgins R, Mcgill I, Molenaar F, et al. Disease risk analysis and post-release health surveillance for a reintroduction programme: the pool frog Pelophylax lessonae. Transbound Emerg Dis. 2017;64(5):1530–48. 27393743 10.1111/tbed.12545

[19] Griffith B, Scott J, Carpenter J, Reed C. Animal translocations and potential disease transmission. J Zoo Wildl Med. 1993;24.

[20] Beckmann KM, Cromie RL, Sainsbury AW, Hilton GM, Ewen JG, Soorae PS, et al. Wildlife health outcomes and opportunities in conservation translocations. Ecol Solut Evid [Internet]. 2022 [cited 2024 Apr 14];3. Available from: https://besjournals.onlinelibrary.wiley.com/doi/10.1002/2688-8319.12164

[21] Warne RK, Chaber AL. Assessing disease risks in wildlife translocation projects: a comprehensive review of disease incidents. Animals. 2023;13. 10.3390/ani13213379 PMC10649731 37958133

[22] Jakob-Hoff Richard, IUCN Species Survival Commission. International Office of Epizootics. Manual of procedures for wildlife disease risk analysis. World Organisation for Animal Health; 2014. 149 p.

[23] Hartley M, Sainsbury A. Methods of disease risk analysis in wildlife translocations for conservation purposes. Ecohealth [Internet]. 2017 Mar 1 [cited 2024 Apr 15];14(1):16–29. Available from: https://link.springer.com/article/10.1007/s10393-016-1134-8 27287192 10.1007/s10393-016-1134-8 PMC5357272

[24] Verant ML, Wolf TM, Romanski MC, Moore S, Mayer T, Munderloh UG, et al. Practical application of disease risk analysis for reintroducing gray wolves (Canis lupus) to Isle Royale National Park, USA. Conserv Sci Pract [Internet]. 2022 Nov 1 [cited 2024 Apr 15];4(11):e12814. Available from: https://onlinelibrary.wiley.com/doi/full/10.1111/csp2.12814

[25] Lees CM, Rutschmann A, Santure AW, Beggs JR. Science-based, stakeholder-inclusive and participatory conservation planning helps reverse the decline of threatened species. Biol Conserv. 2021 Aug 1;260.

[26] Miller RS, Sweeney SJ, Slootmaker C, Grear DA, Salvo PA Di, Kiser D, et al. Cross-species transmission potential between wild pigs, livestock, poultry, wildlife, and humans: implications for disease risk management in North America OPEN. Sci Rep [Internet]. 2017 [cited 2024 Apr 15];7(1):1–14. Available from: www.nature.com/scientificreports/28798293 10.1038/s41598-017-07336-z PMC5552697

[27] Nunn M, Black PF, Murray JG, Nunn MJ. Managing animal disease risk in Australia: The impact of climate change. Rev sci tech Off int Epiz [Internet]. 2008 [cited 2024 Apr 14];27(2):563–80. Available from: https://www.researchgate.net/publication/23285591 18819678

[28] Vaughan-Higgins RJ. Disease risk analysis for wildlife translocation. In: Vogelnest L, Portas T, editors. Medicine of Australian mammals, current veterinary therapy. Melbourne: CSIRO Publishing; 2019.

[29] Woods R, Reiss A, Cox-Witton K, Grillo T, Peters A. The importance of wildlife disease monitoring as part of global surveillance for zoonotic diseases: the role of Australia. Trop Med Infect Dis. 2019;4(1):29. doi: 10.3390/tropicalmed4010029 30736323 PMC6473821

[30] Myers N, Mittermeler RA, Mittermeler CG, Da Fonseca GAB, Kent J. Biodiversity hotspots for conservation priorities. Nature. 2000 Feb 24 [cited 2024 Apr 15];403(6772):853–8. Available from: https://www.nature.com/articles/35002501 10706275 10.1038/35002501

[31] Ceballos G, Ehrlich PR, Barnosky AD, García A, Pringle RM, Palmer TM. Accelerated modern human-induced species losses: entering the sixth mass extinction. Sci Adv. 2015;1(5):e1400253. doi: 10.1126/sciadv.1400253 26601195 PMC4640606

[32] Zamboni T, Di Martino S, Jiménez-Pérez I. A review of a multispecies reintroduction to restore a large ecosystem: the Iberá rewilding program (Argentina). Perspect Ecol Conserv. 2017;15(4):248–56.

[33] Di Bitetti MS, Di Bitetti MS, Di Bitetti MS, Mata J, Svenning JC. Exotic mammals and rewilding in the neotropics. Vol. 29, Mastozoologia Neotropical. SAREM Sociedad Argentina para el Estudio de los Mamiferos; 2022.

[34] Di Martino S, Heinonen S, Donadío E, Fundación Rewilding Argentina. REWILDING EN LA ARGENTINA [Internet]. 1st ed. Lorena López, editor. Buenos Aires: The Conservation Land Trust Argentina; 2022 [cited 2024 Apr 18]. Available from: https://www.rewildingargentina.org/biblioteca/

[35] Guerisoli M, Schiani MI, Teta P, Valenzuela AEJ, Mirol P, Defossé GE, et al. REFLEXIONES ACERCA DEL “REASILVESTRAMIENTO” EN LA ARGENTINA. Vol. 30, Mastozoologia Neotropical. SAREM Sociedad Argentina para el Estudio de los Mamiferos; 2023.

[36] Guerisoli M de las M, Schiaffini MI, Teta P, Valenzuela AEJ, Mirol P, Nori J, et al. Threatened conservation scientists: the aftermath of an eye-opening publication on rewilding. Biol Conserv. 2023 Nov 1;287.

[37] Fundación Rewilding Argentina. Crónicas del Rewilding (Blog). 2022. Los Tapires y el mal de las caderas en Iberá.

[38] Eberhardt AT, Monje LD, Zurvera DA, Beldomenico PM. Detection of Trypanosoma evansi infection in wild capybaras from Argentina using smear microscopy and real-time PCR assays. Vet Parasitol. 2014;202(3–4):226–33. doi: 10.1016/j.vetpar.2014.02.043 24636712

[39] Pinder L, Grosse AP. MAMMALIAN SPECIES Blastocerus dichotomus. 1991 [cited 2024 Apr 15];(380):1–4. Available from: https://academic.oup.com/mspecies/article/doi/10.2307/3504311/2600349

[40] Pinder L, Seal US. Population and habitat viability assessment report for marsh deer Blastocerus dichotomus (PHVA). 1995.

[41] Schaller GB, Manuel J, Vasconcelos C. A marsh deer census in Brazil. Oryx [Internet]. 2018 [cited 2024 Apr 15]; Available from: doi: 10.1017/S0030605300015921S

[42] Duarte JMB, Varela D, Piovezan U, Beccaceci M, Garcia JE. Blastocerus dichotomus, Marsh Deer. The IUCN Red List of Threatened Species [Internet]. 2016; Available from: 10.2305/IUCN.UK.2016-1.RLTS.T2828A22160916.en

[43] Pereira J, Varela D, Aprile G, Cirignoli S, Orozco MM, Lartigau B, et al. Blastocerus dichotomus. In: SAyDS, SAREM, editors. Categorización 2019 de los mamíferos de Argentina según su riesgo de extinción Lista Roja de los mamíferos de Argentina. Buenos Aires; 2019.

[44] MAyDS. Ministerio de Ambiente y Desarrollo Sostenible. Resolución 316/2021. www.argentina.gob.ar, Resolución 316/2021. Argentina: www.argentina.gob.ar; 2021.

[45] Orozco M, Marull C, Jiménez I, Gürtler R. Winter mortality of marsh deer (Blastocerus dichotomus) in wetlands of northeastern Argentina. Mastozool Neotrop. 2013;20:163–70.

[46] Orozco MM, Argibay HD, Minatel L, Guillemi EC, Berra Y, Schapira A, et al. A participatory surveillance of marsh deer (Blastocerus dichotomus) morbidity and mortality in Argentina: first results. BMC Vet Res [Internet]. 2020 Sep 1 [cited 2024 Apr 15];16(1):1–18. Available from: https://link.springer.com/articles/10.1186/s12917-020-02533-x 32873288 10.1186/s12917-020-02533-x PMC7465331

[47] DeAngelo C, DiGiacomo A, JimenezPerez I. Situación poblacional del ciervo de los pantanos Blastocerus dichotomus en los Esteros del Iberá. Rev Mus La Plata Secc Zool. 2011;18(37).

[48] Administración de Parques Nacionales (APN). Sistema de Información de Biodiversidad. http://www.sib.gob.ar. Sistema de Información de Biodiversidad. http://www.sib.gob.ar. 2024.

[49] Quiroga VA, Di Blanco YE, Noss A, Paviolo AJ, Di Bitetti MS, Andrea Quiroga V. The Giant Armadillo (Priodontes maximus) in the Argentine Chaco. Mastozool Neotrop [Internet]. 2017;24(1):163–75. Available from: http://www.sarem.org.arhttp://www.sbmz.com.brhttp://www.sarem.org.ar-http://www.sbmz.com.br

[50] Cerón G, Serrano A, Rosas AC, Vallejos JP, Di Martino S. Fundación Rewilding Argentina. Reintroducción del ciervo de los pantanos en el interfluvio Bermejo-Bermejito (Chaco, Argentina). 2022 [cited 2024 Apr 18]. Available from: https://www.rewildingargentina.org/wp-content/uploads/2023/01/Reintroduccion-del-ciervo-de-los-pantanos-en-el-interfluvio-Bermejo-Bermejito-Chaco.pdf

[51] Burkart R; BN; SR& GDA. Eco-Regiones de la Argentina. Administración de Parques Nacionales. Programa de Desarrollo Institucional Ambiental, Presidencia de la Nación, Administración de Parques Nacionales, editors. Buenos Aires; 1999.

[52] Browne M, Turbek S, Pasian C, Di Giacomo A. Low reproductive success of the endangered Iberá seedeater in its only known breeding site, the Iberá wetlands, Argentina. Ornithol Appl. 2021;123(1).

[53] Ortega L, Miller J, Araguás-Araguás L, Zabala M, Vives L, Mira A. Unravelling groundwater and surface water sources in the esteros del Iberá wetland area: An isotopic approach. Sci Total Environ. 2022;846. 10.1016/j.scitotenv.2022.157475 35868394

[54] Neiff J. El Iberá,¿en peligro? [Internet]. 2004. Available from: www.vidasilvestre.org.ar

[55] Caserta LC, Martins M, Butt SL, Hollingshead NA, Covaleda LM, Ahmed S. White-tailed deer (Odocoileus virginianus) may serve as a wildlife reservoir for nearly extinct SARS-CoV-2 variants of concern. Proc Natl Acad Sci U S A. 2023;120(6). 10.1073/pnas.2215067120 PMC9963525 36719912

[56] Feng A, Bevins S, Chandler J, DeLiberto T, Ghai R, Lantz K. Transmission of SARS-CoV-2 in free-ranging white-tailed deer in the United States. Nat Commun. 2023;14(1). 10.1038/s41467-023-39782-x PMC10333304 37429851

[57] Kotwa JD, Lobb B, Massé A, Gagnier M, Aftanas P, Banerjee A. Genomic and transcriptomic characterization of delta SARS-CoV-2 infection in free-ranging white-tailed deer (Odocoileus virginianus). iScience. 2023;26(11). 10.1016/j.isci.2023.108319 PMC10665813 38026171

[58] Prepelitchi L. Ecoepidemiología de Fasciola hepatica (Trematoda, Digenea) en el norte de la provincia de Corrientes destacando aspectos ecológicos de Lymnaea columella (Pulmonata, Lymnaeidae) y su rol como hospedador intermediario [Internet] [Doctoral disertation]. [Buenos Aires]: Universidad de Buenos Aires; 2009 [cited 2024 Apr 18]. Available from: https://bibliotecadigital.exactas.uba.ar/download/tesis/tesis_n4546_Prepelitchi.pdf

[59] Putman R. The natural history of deer. Cristopher Helm, editor. London: Cornell University Press; 1988.

[60] Polegato B, Zanetti E dos S, Duarte J. Monitoring ovarian cycles, pregnancy and post-partum in captive marsh deer (Blastocerus dichotomus) by measuring fecal steroids. Conserv Physiol. 2018;6(1). 10.1093/conphys/cox073 PMC5786210 29383254

[61] Zanetti E, Duarte J. Reprodução e obstetricia em cervídeos neotropicais. In: Cubas ZS, Silva JCR, Dias JLC, editors. Tratado de animais selvagens: medicina veterinária. 2nd ed. Sao Paulo: Roca. 2014. p. 2519–37.

[62] United Nations. United Nations decade on ecosystem restoration 2021-2030. United Nations Decade on Ecosystem Restorarion. 2019. Available from: https://documents.un.org/doc/undoc/gen/n19/060/20/pdf/n1906020.pdf https://w.w.w.decadeonrestoration.org.

[63] Gerhold R, Hickling G. Diseases associated with translocation of captive cervids in North America. Wildl Soc Bull. 2016;40(1):25–31.

[64] Höfle U, Vicente J, Nagore D, Hurtado A, Peña A, La Fuente J. The risks of translocating wildlife: pathogenic infection with Theileria sp. and Elaeophora elaphi in an imported red deer. Vet Parasitol. 2004;126(4):387–95. 15567043 10.1016/j.vetpar.2004.07.026

[65] Dickens MJ, Delehanty DJ, Michael Romero L. Stress: an inevitable component of animal translocation. Biol Conserv. 2010;143(6):1329–41. doi: 10.1016/j.biocon.2010.02.032

[66] Beringer J, Hansen LP, Wilding W, Fischer J. Factors affecting capture myopathy in white-tailed deer [Internet]. Vol. 60. 1996. Available from: http://www.jstor.orgURL:http://www.jstor.org/stable/3802238

[67] Cattet M, Boulanger J, Stenhouse G, Powell RA, Reynolds-Hogland MJ. An evaluation of long-term capture effects in ursids: implications for wildlife welfare and research [Internet]. 2008. Available from: https://academic.oup.com/jmammal/article/89/4/973/872341

[68] Montané J, Marco I, Manteca X, López J, Lavín S. Delayed acute capture myopathy in three roe deer. J Vet Med A Physiol Pathol Clin Med. 2002;49(2):93–8. doi: 10.1046/j.1439-0442.2002.jv409.x 11958473

[69] Wingfield J. Adrenocortical responses to stress and their modulation in free‐living vertebrates. Compr Physiol. 2010.

[70] McEwen B. Protective and damaging effects of stress mediators. N Engl J Med. 1998;338(3):171–9. 9428819 10.1056/NEJM199801153380307

[71] Guillemi EC, Orozco MM, Argibay HD, Farber MD. Evidence of ehrlichia chaffeensis in argentina through molecular detection in marsh deer (Blastocerus dichotomus). Int J Parasitol Parasites Wildl. 2019;8:45–9. 30619709 10.1016/j.ijppaw.2018.12.004 PMC6312859

[72] Tomassone L, Nuñez P, Gürtler RE, Ceballos LA, Orozco MM, Kitron UD, et al. Molecular detection of Ehrlichia chaffeensis in Amblyomma parvum ticks, Argentina. Emerg Infect Dis. 2008;14(12):1953–5. doi: 10.3201/eid1412.080781 19046533 PMC2634635

[73] Beldomenico PM, Begon M. Stress-host-parasite interactions: a vicious triangle? Revista FAVE-Sección Ciencias Veterinarias [Internet]. 2015 [cited 2024 Apr 14];14:6–19. Available from: 10.14409/favecv.v14i1/3.5160

[74] Wolinska J, King K. Environment can alter selection in host–parasite interactions. Trends Parasitol. 2009;25(5):236–44. 19356982 10.1016/j.pt.2009.02.004

[75] Murray DL, Cox EW, Ballard WB, Whitlaw HA, Lenarz MS, Custer TW, et al. Pathogens, nutritional deficiency, and climate influences on a declining moose population. Wildlife Monographs [Internet]. 2006 [cited 2024 Apr 15];166(1):1–30. Available from: https://wildlife.onlinelibrary.wiley.com/doi/10.2193/0084-0173

[76] Carbillet J, Hollain M, Rey B, Palme R, Pellerin M, Regis C. Age and spatio-temporal variations in food resources modulate stress-immunity relationships in three populations of wild roe deer. Gen Comp Endocrinol. 2023;330:114141. 36272446 10.1016/j.ygcen.2022.114141

[77] Woloshin S, Patel N, Kesselheim AS. False Negative Tests for SARS-CoV-2 infection — Challenges and implications. New Eng J Med [Internet]. 2020 Aug 6 [cited 2024 Apr 15];383(6):e38. Available from: https://www.nejm.org/doi/full/10.1056/NEJMp2015897 10.1056/NEJMp2015897 32502334

[78] Holmes G, Marriott K, Briggs C, Wynne-Jones S. What is rewilding, how should it be done, and why? A Q-method Study of the views held by european rewilding advocates. Conservat Soc. 2020;18(2):77. doi: 10.4103/cs.cs_19_14

[79] Hughes F, Stroh P, Adams W, Kirby K, Mountford J, Warrington S. Monitoring and evaluating large-scale, “open-ended” habitat creation projects: a journey rather than a destination. J Nat Conserv. 2011;19(4):245–53.

[80] Lorimer J, Sandom C, Jepson P, Doughty C, Barua M, Kirby KJ. Rewilding: science, practice, and politics. Annu Rev Environ Resour. 2015;40:39–62.

[81] Reif MK, Theel HJ. Remote sensing for restoration ecology: application for restoring degraded, damaged, transformed, or destroyed ecosystems. Integr Environ Assess Manag. 2017;13(4):614–30. doi: 10.1002/ieam.1847 27627787

[82] Prach K, Durigan G, Fennessy S, Overbeck GE, Torezan JM, Murphy SD. A primer on choosing goals and indicators to evaluate ecological restoration success. Restor Ecol. 2019;27(5):917–23.

[83] Mata JC, Buitenwerf R, Svenning JC. Enhancing monitoring of rewilding progress through wildlife tracking and remote sensing. PLoS One. 2021;16(7). 10.1371/journal.pone.0253148 PMC8270134 34242225

[84] Root-Bernstein M, Galetti M, Ladle R. Rewilding south america: ten key questions. Perspect Ecol Conserv. 2017;15(4):271–81.

[85] Perino A, Pereira H, Navarro L, Fernández N, Bullock J, Ceauşu S, et al. Rewilding complex ecosystems. Science. 2019;364. 10.1126/science.aav5570 31023897

[86] Torres A, Fernández N, Ermgassen S, Helmer W, Revilla E, Saavedra D. Measuring rewilding progress. Philos Trans R Soc B Biol Sci. 2018;373(1761). 10.1098/rstb.2017.0433 PMC6231071 30348877

[87] Nagendra H, Lucas R, Honrado J, Jongman R, Tarantino C, Adamo M. Remote sensing for conservation monitoring: assessing protected areas, habitat extent, habitat condition, species diversity, and threats. Ecol Indic. 2013;33:45–59.

