# Supplementary material for: Applying disease risk analysis for conservation translocations in Argentina: A case study on marsh deer (Blastocerus dichotomus)
Source: PLoS One. 2025 May 20;20(5):e0322878. doi: 10.1371/journal.pone.0322878 (PMC12091794; doi:10.1371/journal.pone.0322878)
Supplement: S2 — These questions will guide you for quantitatively assessing both likelihood of exposure and consequences assigned to each hazard in relation to populations of interest. (DOCX) [file pone.0322878.s002.docx]

**Supporting Information 2. Guiding questions.**

**To evaluate and score the probability of exposure of the two populations of interest (translocated deer and Impenetrable wildlife)** to each disease as Negligible (1), Low (2), Medium (3) or High (4), we can use the excel table and the following guiding questions (we know that some of them have no answer so far for our populations of interest, and in the absence of information we will choose to decide based on individual criteria and previous experience, remembering that the level of confidence with which we make our decisions will be recorded on another scale, which we can then share and discuss as a group).

**The probability of exposure for the population of interest to each disease will then depend on the following:**

1. **Do we know the prevalence of the pathogen for the population of interest?**: The proportion of infected individuals in a given population can affect the probability of encountering a carrier of the pathogen.

2**. Do we know the infectivity and pathogenicity of the agent?**: The infectivity of an agent can be defined as its ability to infect a host and pathogenicity as its ability to produce disease.

3. **Do we know the pathogen's ability to survive in different environments, in organic matter, its incubation time and its level of infectivity?** These factors can favour the probability of exposure.

4. **Is there close contact between individuals or is the species under study gregarious?:** The level of close contact with infected individuals may increase the likelihood of exposure. Situations where feeding sites, shelter areas, latrine areas or other surfaces are shared could increase exposure.

5**.How the pathogen is transmitted?:** How the pathogen is transmitted can influence how and to what extent other individuals may come into contact with it. Direct transmission requires physical contact between the infected and susceptible individual. Indirect transmission occurs through surfaces and objects, or through competent vectors, whose presence may favour greater exposure.

6. **Are there prevention measures in place?:** Prevention measures implemented (generally on domestic species), such as mandatory vaccinations, deworming or sanitary barriers, can significantly reduce the probability of exposure to the pathogen.

**To evaluate and score the consequences of a pathogen on the two populations of interes**t (translocated deer and wildlife of Impenetrable) as Negligible (1), Low (2), Medium (3) or High (4), we can rely on the excel table and the following guiding questions (we know that some of them have no answer so far for our populations of interest, and in the absence of answers we will choose to decide based on individual criteria and previous experience, remembering that the level of confidence with which we make our decisions will be recorded on another scale, which we can then share and discuss as a group):

1.**Do we know the morbidity and/or mortality rate of the pathogen for the population(s) of interest?:** both are important measures to assess the severity of the impact of the pathogen. This may vary by species and pathogen characteristics.

**2. What is the variety or range of species susceptible to become infected and/or diseased with this disease?:** in general terms, if the pathogen affects multiple species (including humans in case of zoonosis or anthropozoonosis), it could have a greater impact and consequences on biodiversity and ecosystems. If it only infects one host without making it sick, the impact could be less.

**3. What is the composition of the receiving environments considering the abundance of generalist species (usually more competent as reservoirs of certain pathogens)?** Consider the dilution effect, for example. Also analyse pathogen specificity, generalism, specialism.

**4. Does the pathogen have an effect on locally threatened species?:** some species may be particularly vulnerable due to small population size, habitat fragmentation or previous threats and may determine the impact of a certain pathogen on the populations under study.

**5. Does the pathogen impact reproduction?:** regardless of mortality rates, pathogens that compromise reproductive fitness and success will have a high impact on the life cycle and sustainability of the populations of interest.

**6. Is it a new pathogen**?: If the pathogen is introduced into a new ecosystem, it could have more dramatic effects due to the lack of previous immunity in the population.
